# Deep active learning-based experimental design to uncover synergistic genetic interactions for host-targeted therapeutics

**DOI:** 10.1093/bioadv/vbaf228

**Published:** 2026-08-28

**Authors:** Haonan Zhu, Mary Silva, Jose Cadena, Braden Soper, Michał Lisicki, Braian Peetoom, Sergio E Baranzini, Shivshankar Sundaram, Priyadip Ray, Jeff Drocco

**Affiliations:** Lawrence Livermore National Laboratory, Livermore, CA 94550, United States; Lawrence Livermore National Laboratory, Livermore, CA 94550, United States; Lawrence Livermore National Laboratory, Livermore, CA 94550, United States; Lawrence Livermore National Laboratory, Livermore, CA 94550, United States; University of Guelph, Ontario, ON N1G 2W1, Canada; Vector Institute, Toronto, ON M5G 0C6, Canada; University of California San Francisco, San Francisco, CA 94158, United States; University of California San Francisco, San Francisco, CA 94158, United States; Lawrence Livermore National Laboratory, Livermore, CA 94550, United States; Lawrence Livermore National Laboratory, Livermore, CA 94550, United States; Lawrence Livermore National Laboratory, Livermore, CA 94550, United States

## Abstract

**Motivation:**

High-throughput methods have advanced the study of host–virus interactions, but testing interactions between host gene pairs during infection remains labor intensive. Identification of multiple gene knockdowns that inhibit viral replication requires exploring a vast combinatorial space and is infeasible via brute-force experiments. Although active learning methods for sequential experimental design have shown promise, existing approaches have generally been restricted to single-gene knockdowns or small-scale double knockdown datasets.

**Results:**

Here, we present an integrated deep active learning (DeepAL) framework that incorporates information from a biological knowledge graph (SPOKE, the Scalable Precision Medicine Open Knowledge Engine) to efficiently search the configuration space of a large dataset of pairwise knockdowns of 356 human genes in HIV infection. Through representation learning, the framework is able to generate task-specific representations of genes while also balancing the exploration-exploitation trade-off to pinpoint highly effective double-knockdown pairs. In addition, we present an ensemble method for improved performance and an interpretation of the gene pairs selected by our algorithm through pathway analysis. To our knowledge, this is the first work to show promising results on double-gene knockdown experimental data of appreciable scale (356 by 356 matrix).

**Availability and implementation:**

https://github.com/LLNL/DeepAL.

## 1 Introduction

Understanding the various pathways essential for viral replication is vital for creating effective antiviral treatments. Due to factors such as replication and mutation rates, many viruses quickly develop resistance to drugs targeting a single site, making them ineffective over time. In these situations, combination therapies are necessary to inhibit viral replication sufficiently to prevent escape through mutations (Tang and Shafer 2012). Although host cellular targets are not subject to the same selective pressures as viral genome components, targeting host factors crucial for viral replication may still benefit from a combination strategy. Viruses have developed intricate interactions with host cells to support their life cycle, and studies have shown that dual knockdowns of host genes can sometimes synergistically inhibit viral growth (Gordon *et al.* 2020). Moreover, targeting multiple host factors may expand the effectiveness of a therapeutic across different viral pathogens (Richman and Nathanson 2016).

Recent technological advances have introduced new high-throughput methods for studying host–virus interactions (Puschnik *et al.* 2017), but testing synergistic interactions between gene pairs during viral infections remains relatively slow and labor-intensive. Identification of promising gene-gene knockdowns requires a search in the combinatorial space of all possible target gene pairs and is infeasible via brute-force experimentation (Norman *et al.* 2019, Replogle *et al.* 2022). A potential solution is sequential experimental design guided by data-driven models to balance the trade-off between successful identification of relevant gene pairs and experimental cost (Sverchkov and Craven 2017). Various approaches, including active learning [e.g. bandits Pacchiano *et al.* (2023)] and traditional experimental design (Lyle *et al.* 2023), have been previously studied in the context of single gene knockdowns. See Mehrjou *et al.* (2021) for an overview of the methods along with benchmark datasets for single genetic interventions. More recently, there has been related work in representation learning to discover pairwise genetic interactions from embeddings of single gene knockdowns (Jain *et al.* 2024a,b), and design of perturbation screens on RNA-seq data by incorporating prior knowledge from multiple data sources (Huang *et al.* 2024a). However, the goal of Jain et al. (2024a, b) is to discover gene pairs that violate the additive assumption (i.e. the effect of a double gene knockdown is the sum of two single gene knockdowns) in double-knockdown experiments, and require measurements from microscopy images while incorporating no existing knowledge graph information to assist in the discovery. To predict the effects of unseen gene knockdown, Huang *et al.* (2024a) operate on RNA-seq data, which have the major drawback that we can only observe perturbations that survive long enough to be measured. There has also been work that utilizes a large language model to design experiments (Huang *et al.* 2024b, Roohani *et al.* 2024). While the initial results are promising, the number of relevant gene pairs that can be identified by the bio-agent is strictly limited due to API constraints and the tendency of large language models to generate hallucinations (i.e. plausible but factually incorrect or nonsensical information) (Xu *et al.* 2024). In summary, although many proposed methods discuss extensions for double-knockdown experimental design, the experimental results from the current literature are limited to single-knockdown data (Huang *et al.* 2024a), small-scale double-knockdown data [e.g. a search space of 50 × 50 matrix (Jain et al. 2024b), only 160 out of 100 576 possible gene pairs are queried (Roohani *et al.* 2024)].

We present an integrated deep active learning (DeepAL) framework that incorporates existing knowledge-graph information to efficiently search the configuration space while balancing the exploration-exploitation tradeoff through an active learning loop that utilizes an ensemble method for an improved predictive model (Lakshminarayanan *et al.* 2017, Abdar et al. 2021). Specifically, we leverage the Scalable Precision Medicine Open Knowledge Engine (SPOKE) (Morris *et al.* 2023). By using graph representation learning (Schlichtkrull *et al.* 2018), our framework is able to provide interpretable results on the set of gene pairs recommended by the framework. This work differs from the previous graph learning-based approach ITERPER Huang *et al.* (2024a) in two ways. First our GNN model is built for heterogeneous knowledge graphs such as SPOKE that include different edge type (gene regulatory interactions, gene-protein encoding, protein-protein interactions, and gene-biological processes) associations whereas the primary graphs used in ITERPER include the gene co-expression graph (a homogeneous graph constructed through thresholding the Pearson correlations) and gene-ontology graph (a bipartite graph between genes and a pathway term constructed through thresholding computed Jaccard indices). Second, unlike ITERPER our work leverages an ensemble method for improved predictive performance which has been studied for deep learning models (Lakshminarayanan *et al.* 2017).

Our main contribution is the development of a unified active learning framework that includes a heterogeneous graph-based representation model that utilizes an existing knowledge graph and an ensemble method for improved predictive performance. To our knowledge, this is the first work to show promising results on double genes knockdown experimental data of appreciable scale (356 by 356 matrix) (Gordon *et al.* 2020).

The rest of the paper is organized as follows: Section 2 introduces the notation and data (SPOKE and HIV dataset) that we use to evaluate our methods, Section 3 presents the proposed ensemble DeepAL framework, Section 4 provides our experimental results on the HIV dataset, and Section 5 summarizes the findings of the article and discusses future directions.

## 2 Notations and data

### 2.1 Notations

We use bold uppercase letters for matrices, bold lowercase letters for vectors, and lowercase letters for scalars. The Hadamard (element-wise) product of vectors a and b is denoted by a°b, and diag denotes the function mapping a vector to a diagonal matrix with the components of the vector as its diagonal entries. We denote the Huber loss function (Huber 1992) by:


(1) $$\text{Huber}\left(a\right)=\{\begin{array}{cc} \frac{1}{2}a^{2} & \text{if}\;|a|\leq 1,\\ |a|-\frac{1}{2} & \text{if}\;|a|>1, \end{array}$$

The activation function Softplus is defined as:


(2) Softplus(a)=log(1+exp(a))

We denote the undirected knowledge graph as G=(V,E,R), where V is the set of vertices, E is the set of edges and R is the set of relationships. Each node $v_{i}\in V$ is associated with a label, label(v)∈L that includes labeled edges. Each edge is represented as a triple $\left(v_{i},r,v_{j}\right)\in E$, where $v_{i}$ and $v_{j}$ are nodes with indices i,j=1,…,|V|, and r∈R denotes the relationship between them. Given a node $v_{i}$, the set of neighbor indices in relation r∈R is denoted as $N_{i}^{r}$. The learned embedding for a given node $v_{i}\in V$ is denoted as $x_{i}\in R^{d}$ where *d* is the pre-assigned embedding dimension. Without loss of generality, we assume that the first *p* nodes are the target genes (e.g. HIV related genes in our experiments), and they are denoted as $H:=\left\{v_{1},\ldots ,v_{p}\right\}\subseteq V$. The pairs of genes of interest are denoted as S:=H×H⊆E, and the viral loads matrix is denoted as $Y\in R^{p\times p}$, where $y_{ij}$ corresponds to the value of the viral loads when we down-regulate the gene $v_{i}$ and the gene $v_{j}$ of *H*.

### 2.2 HIV dataset

Data on the genetic interaction of HIV from Gordon *et al.* (2020) focus on understanding the genetic interactions that influence HIV infection. It includes quantitative measurements of how different genetic perturbations, primarily through CRISPR-based methods, interact to affect viral loads of HIV. In total, the interaction matrix consists of 356 genes prioritized for their established or potential roles in HIV viral replication, as identified through prior studies on host–pathogen interactions and HIV-related protein interactions.

## 3 Methods

**Figure 1:**
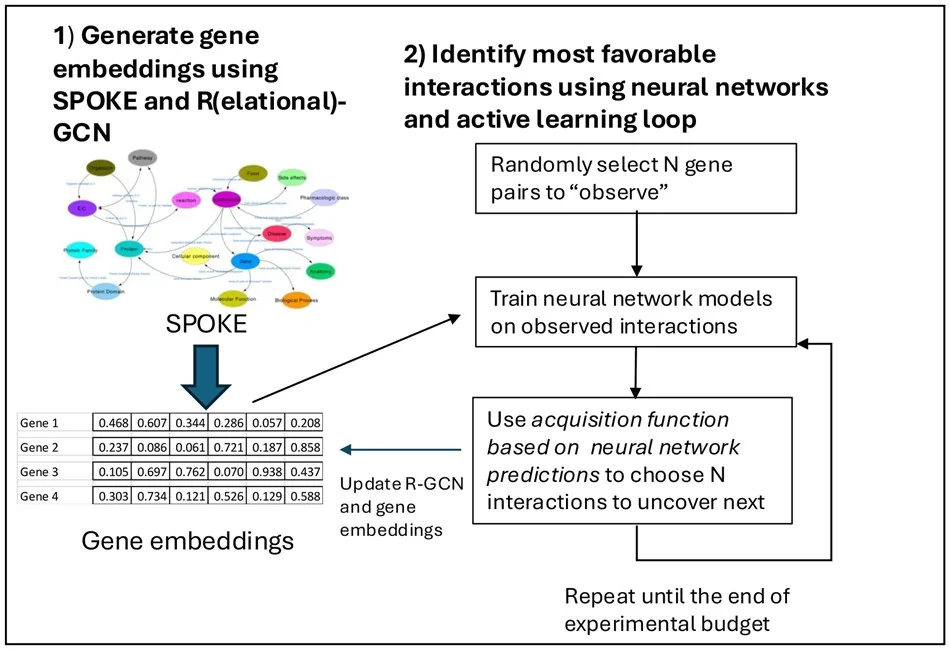
Flowchart of the proposed deep active learning framework.

Our DeepAL framework incorporates information from the knowledge graph to efficiently explore the space of gene pairs. The framework has two phases: (i) initial self-supervised training of *M*-separate representation model(s) with different random initializations to learn the embeddings of genes that summarize the local topology of the knowledge graph (M=1 for the base model), and (ii) an active learning loop where sequentially the representation model(s) and regression model(s) are further optimized to fit the current observed entries of the viral loads matrix followed by the recommendations of the next batch of gene-pairs to uncover. The overall framework is summarized in Fig. 1 and Algorithm 3. In Section 3.2 we summarize the different components of the base model (the representation model and the regression model), in Section 3.3 we discuss improved predictive performance via ensembles, and in Section 3.4 we discuss the acquisition strategy that is developed to balance the exploration-exploitation trade-off.

Algorithm 1 DeepAL

**Require:** knowledge graph G=(V,E,R), set of target genes *H* with p=|H| and collection of gene-pairs of interests *S*

**Require:** queryable viral loads matrix $Y\in R^{p\times p}$ to query

**Require:** number of rounds of experiments *T*, number of gene-pairs to be selected per round *N*

**Require:** R-GCN models $f_{\Theta }^{\left(m\right)}:G\to R^{\left|V\right|\times d},$ where Θ denotes all the learnable parameters from the GNN model, DistMulti weight matrix $R^{\left(m\right)}\in R^{d\times d}$, bilinear regression weight matrices $A^{\left(m\right)}\in R^{d\times d}$ and bias $b^{\left(m\right)}\in R$, where m=1,…,M denotes the distinct models with same architecture

**Require:** Acquisition Strategy, $\Pi :S^{\prime }\to S^{\prime }\subseteq S$ (see section 3.4 for list of strategies considered)

**Optional:** Initial set of gene-pairs $S^{\prime }$ that have already been studied

**1** Train $f_{\Theta }^{\left(m\right)}$ and $R^{\left(m\right)}$ using self-supervised learning (see section 3.2) for each m=1,…,M

**2** If no $S^{\prime }$ is provided, initialize $S^{\prime }$ by randomly drawing *N* pairs of genes from *S*

**3 for**  t=1,…,T//Active Learning Loop

**4 do** 

**5 for**  m=1,…,M//Train each model on  $S^{\prime }$

**6 do** 

**7**  $\min_{\Theta^{\left(m\right)},A^{\left(m\right)},b^{\left(m\right)}}\sum_{(i,j)\in S^{\prime }}\text{Huber}({\hat{y}}_{ij}^{\left(m\right)}-y_{ij})$,

**8**  $S_{\mathrm{new}}\leftarrow \Pi (S^{\prime })$

**9**  $S^{\prime }\leftarrow S^{\prime }\cup S_{\mathrm{new}}$

**return:** observed set of gene-pairs $S^{\prime }\subseteq S$.

### 3.1 SPOKE knowledge graph

The SPOKE knowledge graph (Morris *et al.* 2023) consists of more than 20 000 human gene nodes encoding proteins from Entrez Gene, more than 200 million human proteins, a combined 18 000 nodes of biological processes, molecular function, and cellular components derived from the Gene Ontology database. Additionally, the graph includes over 1 million gene relationships in which the knockdown of one gene, achieved by short hairpin RNA or CRISPR, results in the up-regulation or down-regulation of another gene, as indicated by consensus transcriptional profiles.

To focus on our modeling objectives, we derive a subgraph based on a random walk sampling procedure using the target gene nodes in our viral loads matrix Y as the starting nodes in the random walk. This is effective for excluding nodes that are not closely related to the target genes, and similar ideas have been proposed to learn a meaningful latent representation of social networks (Perozzi *et al.* 2014). The following is a brief description of the random walk algorithm. Denote the subgraph of interest from SPOKE as $G=\left(V^{\prime },E^{\prime },R^{\prime }\right)$, where $V^{\prime }$ contains all the nodes belonging to genes, biological processes, and proteins. For each target gene v∈H, perform w=5 independent random walks of length s=5, i.e. for each walk, we repeatedly select a node from neighbors of the current node with equal probability up to s=5×. This results in a final set of nodes V that lies within the fifth order neighborhood of the target genes *H* and a subgraph G=(V,E,R) where all edges and relationships are restricted to the set of nodes V.

### 3.2 GNN-based model

The proposed DeepAL framework contains three major components: (i) a representation model based on Relational Graph Convolutional Networks (R-GCNs) (Schlichtkrull *et al.* 2018) that maps knowledge graph G to node embeddings ${\left\{x_{i}\right\}}_{v_{i}\in V}$, (ii) an edge prediction model based on DistMulti (Yang et al. 2014) that maps a given pair of embeddings $\left(x_{i},x_{j}\right)$ to a binary prediction of whether the two nodes are related by relationship r∈R, and (iii) a bilinear regression model that maps a pair of node embeddings to the target variable of interest in the active learning loop. The additional model configurations are summarized in Table 1.

**Table 1:** Summary of hyperparameter setups for different components of the framework.

Model	Hyperparameters			
	Normalization	Non-linearlity σ	Regularization	Dimensional parameters
R-GCN	DiffGroupNorm (Zhou *et al.* 2020)	ReLu	Weight decay	*L* = 3^a^
				$d_{h}=64$
				d=50
DistMulti	N/A	Sigmoid	N/A	N/A
Bilinear	N/A	Softplus	Dropout (Srivastava *et al.* 2014)	N/A
			Weight decay	

a This balances the trade-off between expressiveness of the model and the over-smoothing phenomenon where all nodes became indistinguishable in the embedding space Rusch *et al.* (2023).

#### 3.2.1 R-GCN

The main propagation model for the forward-pass of a node $v_{i}$ is:


(3) $$h_{i}^{l+1}=\sigma \left(\sum_{r\in R}\sum_{j\in N_{i}^{r}}\frac{1}{c_{i,r}}W_{r}^{l}h_{j}^{l}\right)+W_{0}^{l}h_{i}^{l},$$

where l=1,…,L indexes the layer number and *L* is the total number of convolution layers. $h_{i}^{l}\in R^{d_{h}}$ denotes the embedding of node *i* at the *l*’th layer, where $d_{h}$ is the dimension of the hidden layers. $c_{i,r}$ is a normalization constant such that $c_{i,r}=\left|N_{i}^{r}\right|$, the number of neighbors of node $v_{i}$ having relationship *r*. Each graph convolution operation aggregates feature vectors of each node’s neighbors. In this work, we use the standard basis vectors for the initial embeddings (i.e. $h_{i}^{0}=e_{i}\in R^{\left|V\right|}$ for all $v_{i}\in V$).

#### 3.2.2 DistMulti: edge prediction model for initialization

Every relation *r* is associated with a diagonal matrix $R_{r}\in R^{d\times d}$, and for every pair of nodes $\left(v_{i},v_{j}\right)$ is scored as:


(4) $$f\left(v_{i},r,v_{j}\right)=\sigma \left(x_{i}^{⊤}R_{r}x_{j}\right).$$

The self-supervised learning task is performed based on negative sampling (Yang *et al.* 2014, Trouillon *et al.* 2016, Schlichtkrull *et al.* 2018) where for each observed relationship (i.e. positive edge), we sample one false edge (i.e. negative edge), and both the representation model and DistMulti are jointly trained to classify these edges.

#### 3.2.3 Bilinear regression

For every pair of target nodes $\left(v_{i},v_{j}\right)\in S$, the predicted viral loads are given by:


(5) $${\hat{y}}_{ij}=\text{Softplus}\left(x_{i}^{⊤}Ax_{j}\right)+b,$$

where A is a trainable symmetric weight matrix and *b* is a trainable scalar for bias correction. This is a generalization of DistMulti, where the matrix is no longer restricted to be diagonal, and an additional nonlinear transformation is used to increase the expressiveness of the model. This bilinear formulation has been extensively studied in the bilinear bandit literature (Jun *et al.* 2019, Rizk *et al.* 2021).

### 3.3 Ensemble method for improving predictive performance

Performance of the predictive model is a critical part of our model-based active learning framework, and the ensemble method has been used with success for neural network-based models in supervised learning settings (Lakshminarayanan *et al.* 2017, Abdar *et al.* 2021). Given *M* trained models, we have *M* predictions [${\hat{Y}}_{ij}^{\left(m\right)},m=1,\ldots ,M$, Equation (5)] for every pair of nodes $\left(v_{i},v_{j}\right)$ that have not been uncovered. These *M* realizations reflect the predictive model uncertainty, and we can compute statistical estimates such as standard deviations and quantiles that are useful for the acquisition strategies discussed in the following subsection.

### 3.4 Acquisition strategies

We examine multiple acquisition strategies for our GNN-based model and the ensemble extension, and they are summarized in Table 2. The acquisition strategies fall into three categories:

**Table 2:** Comparison of acquisition strategies for the GNN-based model and ensemble extension.^a^

Acquisitions	GNN-based model M=1	Ensemble extension M=20
Greedy	$\arg\min_{(i,j)\in S^{\prime }}{\hat{y}}_{ij}$	$\arg\min_{(i,j)\in S^{\prime }}{\text{median}}_{m}{\hat{y}}_{ij}^{\left(m\right)}$
Badge^b^	$\arg\max_{(i,j)\in S^{\prime }}\left\|x_{i}\;○\;x_{j}\right\|$	$\arg\max_{(i,j)\in S^{\prime }}{\text{median}}_{m}\left\|x_{i}^{\left(m\right)}{}^{\circ}x_{j}^{\left(m\right)}\right\|$
Optimism	N/A	$\arg\min_{(i,j)\in S^{\prime }}{\text{quantile}}_{m}{\hat{y}}_{ij}^{\left(m\right)}$
Maximum variance	N/A	$\arg\max_{(i,j)\in S^{\prime }}{\text{std}}_{m}{\hat{y}}_{ij}^{\left(m\right)}$

a Both optimism and maximum variance requires uncertainty quantification hence unattainable in the base model setting.

b First proposed in Ash *et al.* (2019) to use norm of the gradient from the last layer of the neural network for uncertainty quantification. This reduces to T(race)-optimality in optimal experimental design Pukelsheim (2006) when there is no-hidden layers.

1. Exploitation (Greedy): These strategies prioritize the top-performing gene pairs predicted by the current model(s). While effective in identifying strong candidates, they may not explore the broader search space, potentially overlooking other promising gene combinations.
2. Exploration [Badge (Ash *et al.* 2019), Maximum Variance]: These strategies prioritize gene pairs with the highest uncertainty as predicted by the model(s). Although they excel at exploring areas of the search space with the highest uncertainty (leading to increased information gain), they may lead to suboptimal results in terms of identifying the best performing pairs.
3. Balanced (Optimism): These strategies aim to strike a balance between exploitation and exploration, leveraging the strengths of both approaches to achieve a more effective search.

In addition, we have the following model-agnostic acquisition strategies as benchmarks:

1. Uniform Random Sampling: uniformly sample the gene pairs without replacement.
2. Degree-Based Random Sampling: sample the gene pairs proportional (the sampling probability is computed by a softmax transformation of the sums of node degrees) to the sum of their node degrees in the SPOKE knowledge graph (Section 3.1).
3. Path-Based Random Sampling: (i) precompute the shortest path between all gene pairs of interest, (ii) sample without replacement incrementally starting from gene pairs with the shortest path. This approach focuses on gene pairs that are close in the SPOKE knowledge graph.

## 4 Experiments

In this section, we evaluate the performance of our methods on the HIV dataset described in Section 2.2, and our main result shows that by incorporating information from the knowledge graph and utilizing improved predictions through the ensemble approach, the proposed ensemble DeepAL framework uncovers 92% of the top 400 gene-pairs after observing less than 6.3% of the entire matrix.

### 4.1 Performance evaluation

To mimic real-world experimental needs, we run all algorithms for 17 rounds (including the initial round of random selection), and at each round each algorithm uncovers 400 gene pairs for observation, where the batch size of 400 is chosen to approximately balance typical computational and experimental capabilities while allowing sufficient exploration of the interaction matrix. We use Coverage@400 to evaluate our method, and it is defined as the fraction of top-400 gene pairs uncovered cumulatively by the end of each round. This metric assesses how well the active learning framework can uncover the most relevant gene pairs and is our main objective.

### 4.2 Comparison among the acquisition strategies

**Figure 2:**
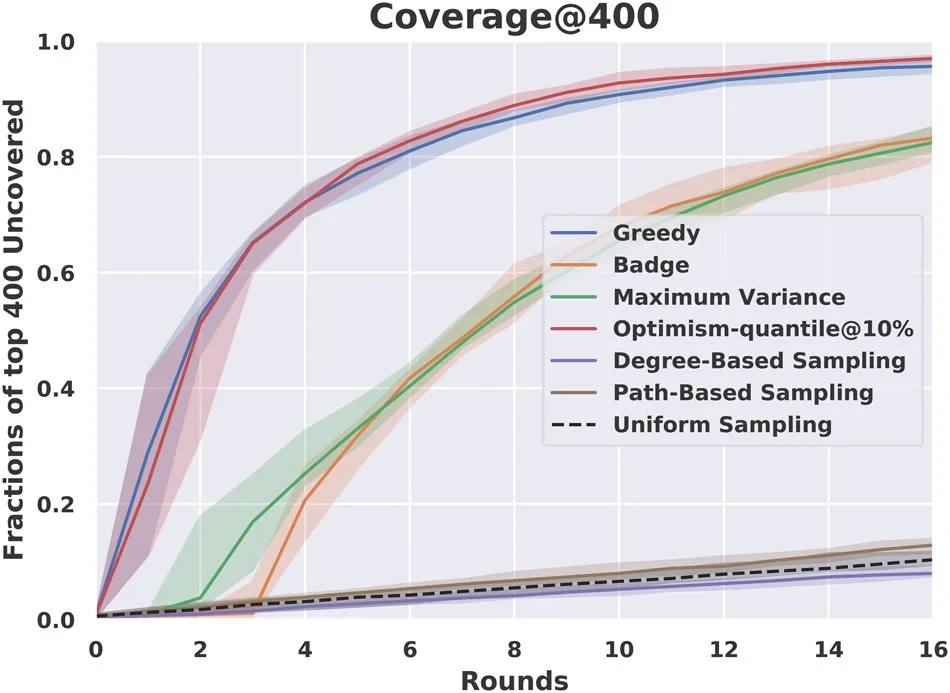
Comparison among the different acquisition strategies, and results are summarized over 20 replicates. Optimism with the 10% quantile achieves the best performance in terms of coverage at terminal phase, and uncovers 92% of the top 400 gene-pairs while only <6.3% of the entire matrix is observed.

We evaluate the list of acquisition functions detailed in Table 2, and the results are summarized in Fig. 2. Optimism with the 10% quantile achieves the best performance in terms of coverage at the end of the active sensing process, while the greedy solution offers competitive performance, especially in the early rounds. Badge and maximum variance strategies are suboptimal for coverage due to an emphasis on exploration over exploitation.

### 4.3 Ablation studies

To demonstrate the significance of the various components of our model, we perform the following ablation studies:

1. DeepAL: GNN-based model.
2. DeepAL-Ensemble: ensemble extension of the proposed model with M=20.
3. DeepAL-Ensemble-Randominit: ensemble of the base models but all the weights are initialized randomly instead of performing self-supervised training through negative sampling.
4. Ensemble of Models with Fixed Features (FF-Ensemble): this is the case where the embeddings are frozen after initial training on the knowledge graph, and the comparison aims to examine the benefits of fine-tuning the embeddings on the observed viral loads data.
5. Ensemble of Models with Unconstrained Features (UF-Ensemble): this is the case where the embeddings are treated as free parameters to be optimized and has been studied extensively to understand behaviors of neural networks (Zhu *et al.* 2021, Mixon *et al.* 2022). The comparison aims to examine the benefits of incorporating knowledge graph information into the model.

**Figure 3:**
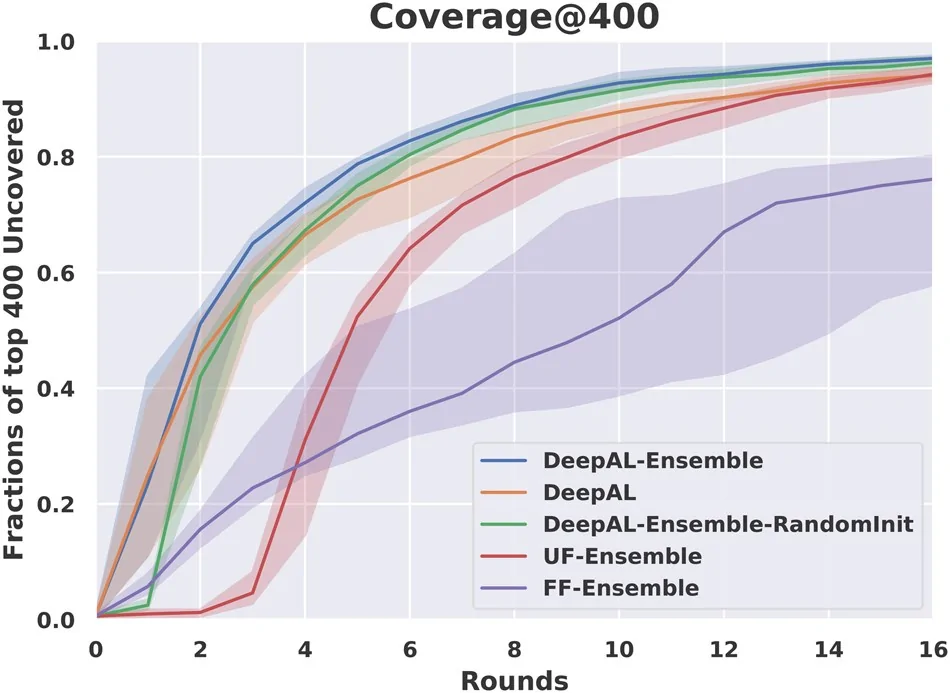
Ablation studies to validate our proposed approach, and results are summarized over 20 replicates. The comparison between the proposed GNN-based model and the ensemble extension shows that there is significant gains in coverage through improved predictive model. The comparison between DeepAL-Ensemble versus UF-Ensemble shows that the graph representation of the models from SPOKE offers a more effective representation of the genes for downstream tasks. The comparison between the DeepAL-ensemble and DeepAL-Ensemble with random initialization shows that there is meaningful information in the SPOKE graph that can benefits the search for best genes in the early rounds, but the benefits diminish rapidly as more experimental data are collected; The comparison between DeepAL-Ensemble and FF-Ensemble shows that there is significant benefits to fine-tuning the embeddings as more data is being collected.

In Fig. 3, we compare our best performing acquisition strategy (optimism with the 10% quantile) for all ensemble-based approaches listed above and a greedy acquisition strategy for the base model. The comparison between the proposed GNN-based model and the ensemble extension shows that there are significant gains in coverage through an improved predictive model. The comparison between DeepAL-Ensemble versus UF-Ensemble shows that the graph embeddings of the models extracted from SPOKE offers a more effective representation of the genes for downstream tasks. The comparison between the DeepAL-ensemble and DeepAL-Ensemble with random initialization shows that there is meaningful information in the SPOKE graph that can accelerate the search for the best genes in the early rounds when only a small amount of experimental data has been observed. The comparison between DeepAL-Ensemble and FF-Ensemble shows that there is significant benefit in fine-tuning embeddings based on observed experimental data.

### 4.4 Pathway analysis

**Figure 4:**
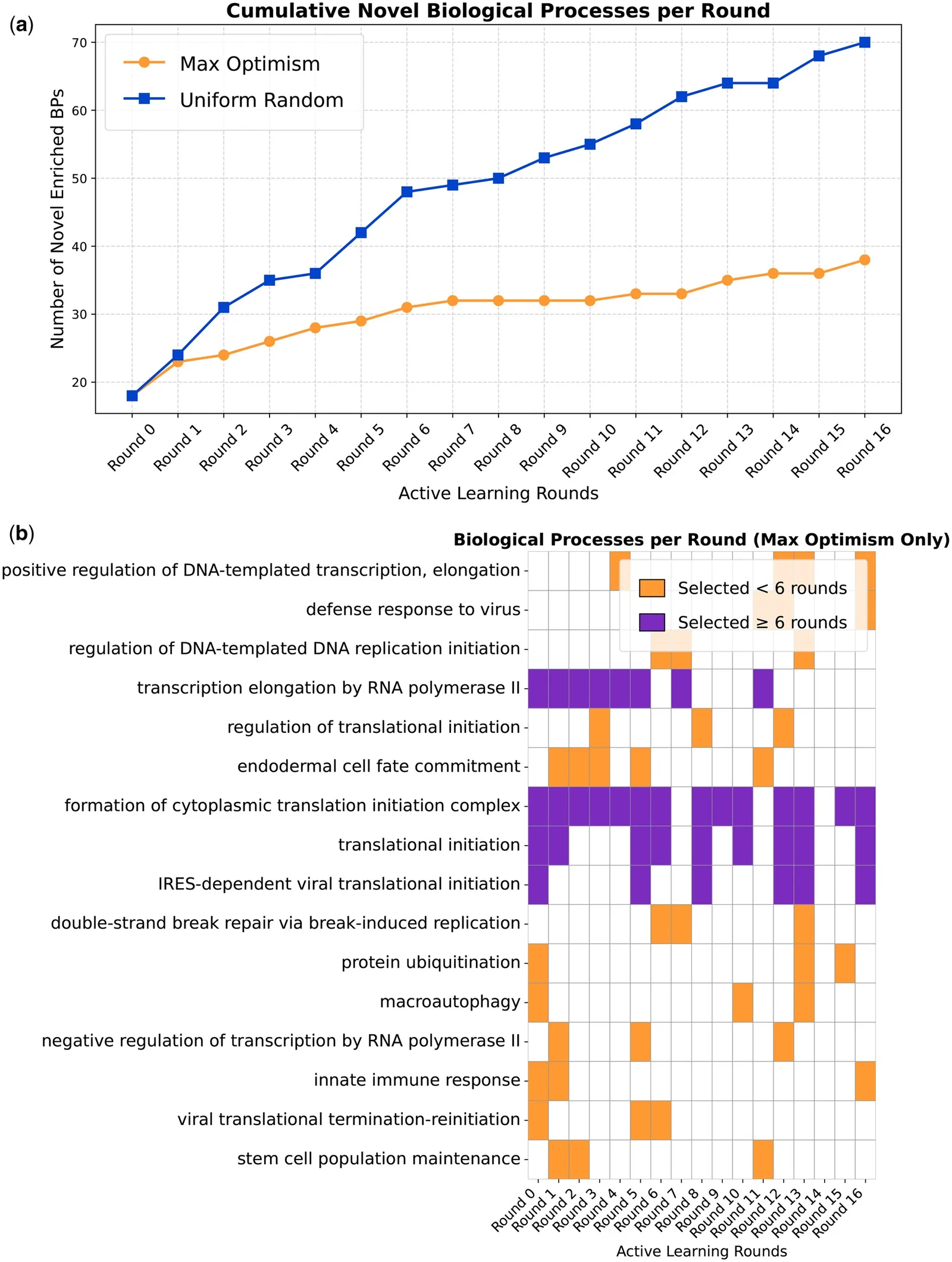
Pathway enrichment analysis of gene pairs selected by DeepAL. (a) Comparison between Optimism with 10% quantile and uniform sampling shows that while the random baseline explores a broader set of biological processes, Optimism with 10% quantile focuses on a more selective subset. (b) Heatmap reveals that Optimism with 10% quantile repeatedly enriches translation- and transcription-related biological processes over multiple rounds, suggesting convergence on biologically relevant pathways.

To provide a biologically intuitive representation of the gene selection process during active learning, we performed a pathway enrichment analysis, with the results summarized in Fig. 4. This analysis involved identifying the gene pairs most frequently selected in 16 independent runs and grouping the top pairs for each round. Using GOATOOLS (Klopfenstein *et al.* 2018) for gene enrichment, each gene group was tested against all coding genes and overrepresented terms were plotted.

In this analysis, we compare the Optimism with the 10% quantile selection approach to a baseline of uniform sampling. Figure 4a shows the cumulative number of novel enriched biological processes (BPs) identified across the experimental rounds. As expected, random sampling identifies a greater total number of novel BPs as a result of its unstructured exploration. However, this comes at the cost of consistency, with many BPs appearing only once and no clear biological focus emerging.

In contrast, Optimism with 10% quantile consistently prioritizes a more selective biologically coherent set of BPs. As shown in Fig. 4b, several processes—particularly those related to translation and transcription—are repeatedly selected in multiple rounds (purple), indicating convergence in pathways likely to be mechanistically relevant for viral replication.

The biological processes enriched by Optimism with 10% quantile exhibit a strong functional theme: many are involved in the translation and processing of RNA. As shown in Fig. 4b, several of these processes such as translational initiation, formation of cytoplasmic translation initiation complex, appear consistently in six or more rounds (purple). This recurrence highlights that Optimism with 10% quantile is able to converge on biologically coherent pathways that are mechanistically linked to HIV replication.

This focus is particularly relevant given that HIV is highly dependent on host translational machinery. For example, translational initiation and transcription elongation by RNA polymerase II are essential for viral protein synthesis. The enrichment of these processes suggests that Optimism with 10% quantile repeatedly identifies genes whose perturbation could disrupt HIV replication at the translational level.

Uniform random sampling does not display this consistency: the biological processes it identifies appear sporadically, with few processes enriched in more than one round. This further supports the interpretation that Optimism with 10% quantile selectively amplifies signal around specific antiviral mechanisms, whereas random sampling lacks biological focus.

## 5 Conclusion

In this work, an integrated DeepAL framework is proposed to incorporate existing heterogeneous knowledge graph information for efficient double-gene knockdown experiment design for host-targeted therapeutics. This unified framework utilizes the expressiveness of graph representation learning, the improved predictive model based on ensemble method, and the sample efficiency of the active learning loop to show promising results on double-gene knockdown experimental data of appreciable scale. In addition, the framework can provide meaningful representations of genes, leading to interpretable results on the set of gene pairs recommended by the framework.

There are several directions for future work. One direction is to integrate pre-trained large language models into the framework for initial recommendation steps, since these pre-trained models have demonstrated the ability to provide valuable information for a warm start; the second direction is to incorporate additional information from the knowledge graph into the model (e.g. node features) to better differentiate the gene nodes; a third direction is to develop an efficient Graph Learning inference method through neighbor subsampling, which enables us to utilize the full knowledge graph into our model instead of a subgraph; A fourth direction is a more thorough investigation on the uncertainty quantification provided by ensemble method through more diverse sets of predictive models.

## Supplementary Material

vbaf228_Supplementary_Data
