# Supplementary material for: Deep active learning-based experimental design to uncover synergistic genetic interactions for host-targeted therapeutics
Source: Bioinform Adv. 2026 Aug 28;6(1):vbaf228. doi: 10.1093/bioadv/vbaf228 (PMC13524425; doi:10.1093/bioadv/vbaf228)
Supplement: Supplementary file 1 [file vbaf228_supplementary_data.zip › Figure2b_supp.pdf]

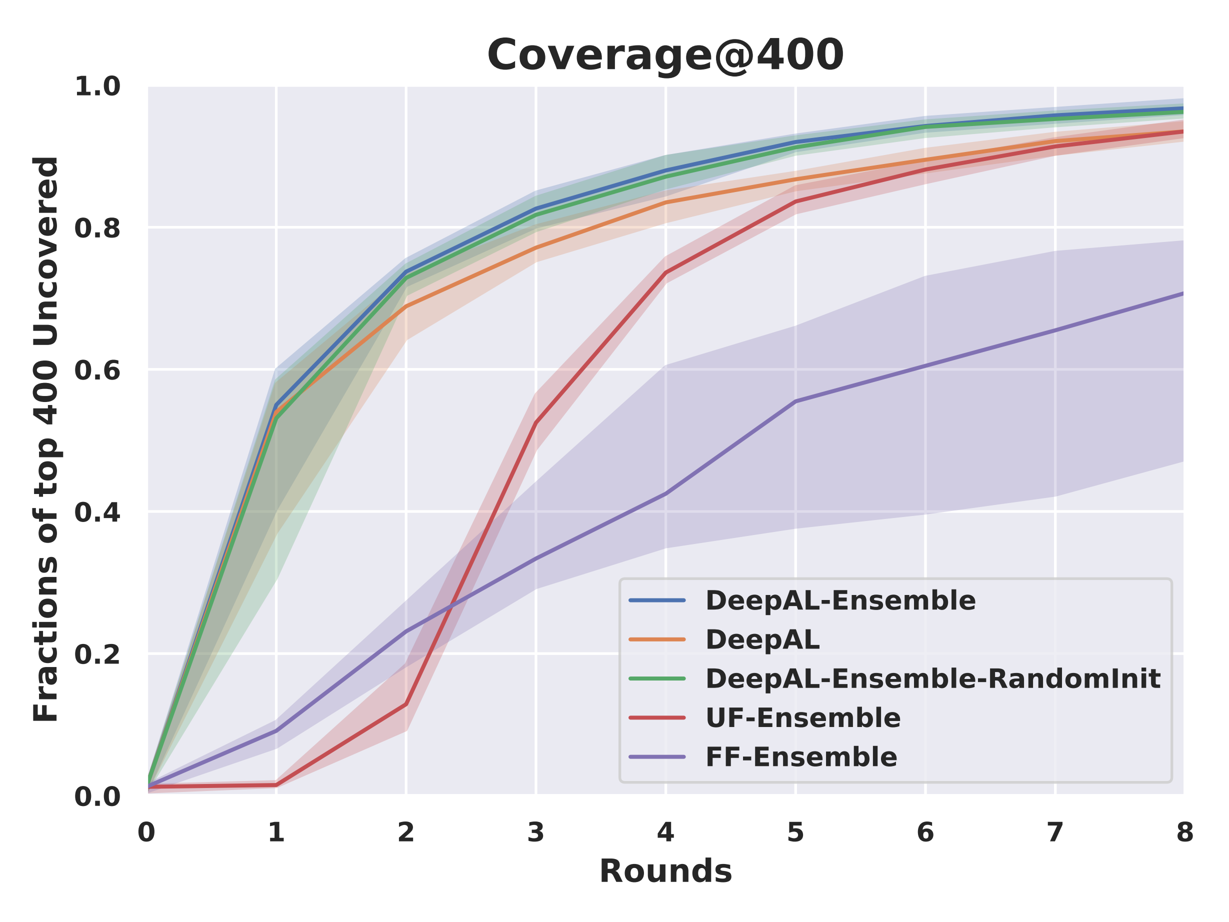
Coverage@400
Fractions of top 400 Uncovered
Rounds
1.0
0.8
0.6
0.4
0.2
0.0
0
1
2
3
4
5
6
7
8
DeepAL-Ensemble
DeepAL
DeepAL-Ensemble-RandomInit
UF-Ensemble
FF-Ensemble
