# Supplementary material for: Deep active learning-based experimental design to uncover synergistic genetic interactions for host-targeted therapeutics
Source: Bioinform Adv. 2026 Aug 28;6(1):vbaf228. doi: 10.1093/bioadv/vbaf228 (PMC13524425; doi:10.1093/bioadv/vbaf228)
Supplement: Supplementary file 1 [file vbaf228_supplementary_data.zip › Deep_Active_Learning__DeepAL__for_Genetic_Interaction_Experimental_Design-supp-final.pdf]

ISMB 2025 Proceeding, 2025, pp. 1–2

doi: DOI
Paper

PAPER

Supplementary: Deep Active Learning based Experimental Design to Uncover Synergistic Genetic Interactions for Host Targeted Therapeutics

Haonan Zhu,[1,*] Mary Silva,[1] Jose Cadena,[1] Braden Soper,[1] Michał Lisicki,[2,3] Braian Peetoom,[4] Sergio E. Baranzini,[4] Shankar Sundaram,[1] Priyadip Ray[1] and Jeff Drocco[1]

[1]Lawrence Livermore National Laboratory, 7000 East Ave, 94550, CA, USA, [2] University of Guelph, 50 Stone Rd E, N1G 2W1, ON, Canada, [3] Vector Institute, 108 College St W1140, M5G 0C6, ON, Canada and [4] University of California San Francisco, 675 Nelson Rising Lane, 94158, CA, USA

*Corresponding author. zhu18@llnl.gov

Abstract

Additional Experimental Results

In this section, we provide additional experimental results on the HIV dataset [1] with different batch size (200 and 800). In the first setting, we run all algorithms for 33 rounds (including the initial round of random selection), and at each round each algorithm selects 200 gene pairs for observation; for the second setting, we run all algorithms for 9 rounds (including the initial round of random selection), and at each round each algorithm selects 800 gene pairs for observation.

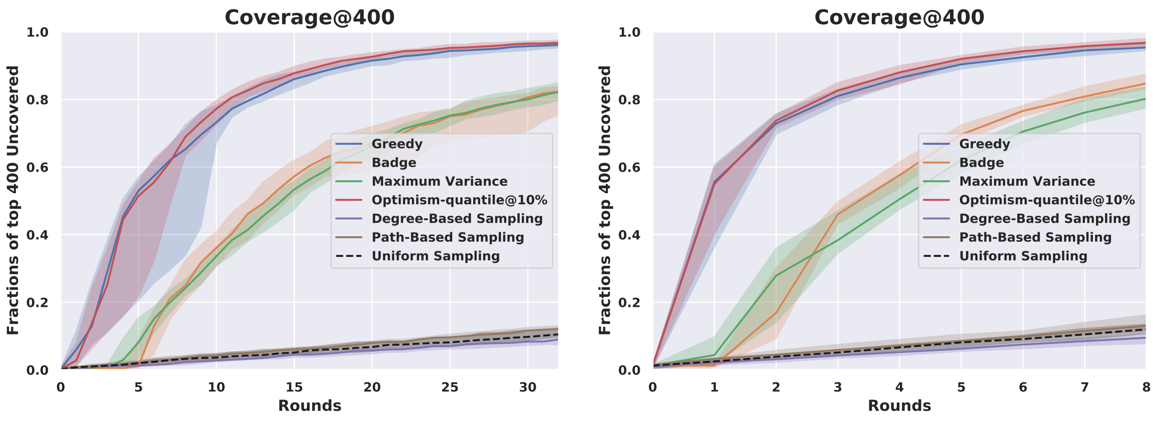

(a) Batch Size=200 (b) Batch Size=800

Figure 1. Comparison among the different acquisition strategies, and results dare summarized over 20 replicates. Optimism with 10% quantiles achieves the best performance in terms of coverage at terminal phase.

© The Author 2025. Published by Oxford University Press. All rights reserved. For permissions, please e-mail: journals.permissions@oup.com

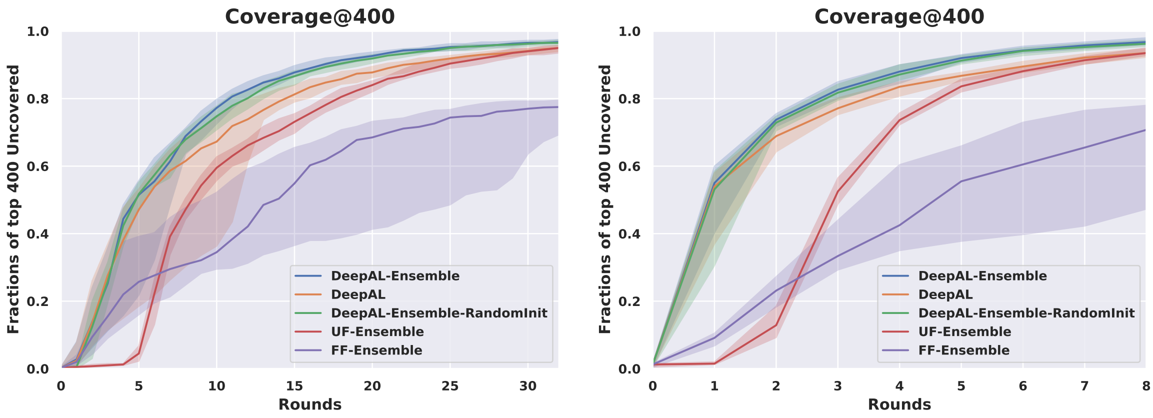

(a) Batch Size=200

(b) Batch Size=800

Figure 2. Abalation studies to validate our proposed approach, and results are summarized over 20 replicates.

References

1. David E Gordon, Ariane Watson, Assen Roguev, Simin Zheng, Gwendolyn M Jang, Joshua Kane, Jiewei Xu, Jeffrey Z Guo, Erica Stevenson, Danielle L Swaney, et al. A quantitative genetic interaction map of hiv infection. *Molecular cell*, 78(2):197–209, 2020.
